# Similar and Differing Distributions Between ^18^F-FDG-PET and Arterial Spin Labeling Imaging in Temporal Lobe Epilepsy

**DOI:** 10.3389/fneur.2019.00318

**Published:** 2019-04-03

**Authors:** Daichi Sone, Norihide Maikusa, Noriko Sato, Yukio Kimura, Miho Ota, Hiroshi Matsuda

**Affiliations:** ^1^Integrative Brain Imaging Center, National Center of Neurology and Psychiatry, Tokyo, Japan; ^2^Department of Radiology, National Center of Neurology and Psychiatry, Tokyo, Japan; ^3^Division of Clinical Medicine, Department of Neuropsychiatry, Faculty of Medicine, University of Tsukuba, Ibaraki, Japan

**Keywords:** temporal lobe epilepsy, 18F-FDG PET, arterial spin labeling, cerebral blood flow, cerebral glucose metabolism

## Abstract

**Background:** Despite the increasing use of arterial spin labeling (ASL) in patients with epilepsy, little is known about its brain regional distribution pattern, including diaschisis, and its correspondence with FDG-PET. Here, we investigated the regional match and mismatch between FDG-PET and ASL in temporal lobe epilepsy (TLE).

**Methods:** We recruited 27 patients with unilateral TLE, who underwent inter-ictal ASL and FDG-PET scans. These images were spatially normalized using Statistical Parametric Mapping 12, and the regional values in both ASL and FDG-PET were calculated using PMOD software within 20 volumes of interest (VOIs), including the temporal lobe, adjacent cortices, subcortical structures, and cerebellum. ASL images of 37 healthy controls were also analyzed and compared.

**Results:** Whereas, ASL showed significant side differences, mainly in the temporal and frontal lobes, the significant abnormalities in FDG-PET were more widespread and included the insula and supramarginal gyrus. Ipsilateral thalamic reduction was found in FDG-PET only. The detectability of the focus side compared with the contralateral side was generally higher in FDG-PET. The discriminative values in ASL compared with healthy controls were higher in temporal neocortex and amygdala VOIs.

**Conclusions:** There are similar and differing regional distributions between FDG-PET and ASL in TLE, possibly reflecting regional match and mismatch of cerebral blood flow and metabolism. At this stage, it seems that ASL couldn't present comparable clinical usefulness with FDG-PET. These findings deepen our knowledge of ASL imaging and are potentially useful for its further application.

## Introduction

Temporal lobe epilepsy (TLE) is the most common focal epilepsy in adults and often causes pharmacoresistant seizures (1). Clear evidence shows that neurosurgical resection is more recommendable in refractory TLE than continued use of anti-epileptic drugs (2), and precise lateralization of the focus side is thus important in clinical practice. The current presurgical examinations of focal epilepsy typically involve multimodal neuroimaging, including high-resolution MRI, ^18^F-fluorodeoxyglucose positron emission tomography (FDG-PET), and perfusion single-photon emission computed tomography (SPECT), as well as neurophysiological tests such as electroencephalography (EEG) and magnetoencephalography (MEG) (3).

Although FDG-PET is a well-established modality with high sensitivity of 85–90% for detecting the focal hypometabolism in TLE (4), it has several drawbacks, such as radiation exposure, high cost, and limited availability in developing countries (5). Accordingly, perfusion MRI, that is, arterial spin labeling (ASL) imaging, is expected to be useful in noninvasive detection of inter-ictal hypoperfusion on the focus side in TLE. Indeed, a growing number of studies have reported the usefulness of ASL for detecting the seizure focus (6–14). Additionally, ASL is widely applied to other types of studies in epilepsy, such as those of cerebral blood flow (CBF) networks (15), relationships between CBF and brain temperature (16), and idiopathic generalized epilepsy (17).

On the other hand, inter-ictal perfusion imaging, such as SPECT, has conventionally been regarded as a less sensitive examination than FDG-PET or ictal perfusion (4). Furthermore, there is evidence of a significant regional mismatch between glycometabolism and CBF, which leads to a different detectability of the focus in TLE (18). As for the regional distribution of FDG-PET, inter-ictal hypometabolic areas spread to ipsilateral fronto-temporal cortices, the thalamus, and other areas in TLE (4). Additionally, contralateral cerebellar hypometabolism, that is, crossed-cerebellar diaschisis (CCD), can be seen in focal epilepsies (4). However, little is known about the brain regional distribution of inter-ictal hypoperfusion in ASL or about the similarities and differences between FDG-PET and ASL. Considering the increasing application of ASL to epilepsy, it would be relevant to deepen our understanding of this aspect.

To demonstrate the regional match and mismatch between FDG-PET and ASL in TLE, this study used volume of interest (VOI) analyses to investigate (1) regional side differences and (2) the detectability of the focus in each region.

## Methods

### Subjects

We recruited 27 patients with unilateral TLE (14 women, 13 men; mean ± SD age, 38.2 ± 13.2 years) at our epilepsy center between July 2016 and October 2017. TLE was diagnosed by board-certified epileptologists based on the presence of focal seizures consistent with TLE and focal epileptiform discharge predominantly in temporal areas on conventional scalp EEG. All patients underwent 3T-MRI and inter-ictal FDG-PET, which were visually evaluated by experienced neuroradiologists. Clinical data were also reviewed and included age at seizure onset, seizure types, anti-epileptic drugs, and long-term video-EEG monitoring. The clinical demographics are detailed in the Results section.

Patients with the following criteria were excluded: a significant medical history of acute encephalitis, meningitis, severe head trauma, or ischemic encephalopathy; suspicious epileptogenic lesions (e.g., tumor, cortical dysplasia, or vascular malformation) on MRI other than ipsilateral HS; contradictory lateralization of focus among MRI, FDG-PET, and long-term video-EEG monitoring; or epileptic paroxysms in extratemporal regions on EEG.

We also recruited 37 healthy individuals as controls for ASL imaging (21 women, 16 men; mean ± SD age, 39.6 ± 12.4 years). There were no significant differences between the two groups in terms of age (*p* = 0.682, unpaired *t*-test) or sex (*p* = 0.697, chi-square test). All participants provided written informed consent, and this study was approved by the Institutional Review Board at the National Center of Neurology and Psychiatry Hospital.

### MRI Acquisition and Processing

MR imaging was performed on a 3-T MR system (Philips Medical Systems, Best, The Netherlands). We confirmed that the patients had no seizures within the 24 h prior to the examination by interview, and no seizures were observed during the scan.

The CBF estimation was performed using the three-dimensional (3D) pseudo-continuous ASL (pCASL) technique. The imaging parameters were single-shot gradient-echo echo planar imaging in combination with parallel imaging (sensitivity encoding factor, 2.0); repetition time (TR)/echo time (TE), 5,716/20.5 ms; matrix, 80 × 80; field of view (FOV), 24 × 24 cm; slice thickness, 3 mm with no gap; 52 slices; labeling duration, 1,650 ms; postspin labeling delay, 1,800 ms; no time interval between consecutive slice acquisitions; radio frequency duration, 0.7 ms; pause between radio frequency pulses, 0.7 ms; labeling pulse flip angle, 25°; bandwidth, 2.2 kHz/pixel; and echo train length, 100. Four pairs of control/label images were acquired and averaged. For measurement of the magnetization of arterial blood and for segmentation purposes, an echo planar imaging M0 image was obtained separately with the same geometry and the same imaging parameters as the pCASL but without labeling.

We also added 3D T1-weighted image and other routine MRI sequences for TLE with the following sequences: 3D T1-weighted images (TR/TE, 7.18 ms/3.46 ms; flip angle, 10°; effective slice thickness, 0.6 mm with no gap; 300 slices; matrix, 384 × 384; FOV, 26.1 × 26.1 cm); high-resolution T2-weighted images (TR/TE, 6,000/78 ms; flip angle, 90°; 0.43 × 0.43 mm^2^ in-plane resolution; slice thickness, 2 mm with no gap; 32 slices; matrix, 476 × 377; FOV, 22 × 24 cm); and 3D fluid-attenuated inversion recovery (FLAIR) images (TR/TE, 4,700/283 ms; inversion time, 1,600 ms; thickness, 0.55 mm with no gap; 340 slices; matrix, 512 × 465; FOV, 26 × 23.4 cm).

The data collected from the pCASL were analyzed using ASLtbx software working on MATLAB (MathWorks, Natick, MA) (19). Individual CBF images contained some patchy noise and therefore a simple median filter (a nonlinear digital filtering technique) with 3 × 3 voxels was used, as in our previous studies (15–17). These images were spatially normalized using the DARTEL (diffeomorphic anatomical registration using the exponentiated lie) method (20) and Statistical Parametric Mapping 12 (SPM12; http://www.fil.ion.ucl.ac.uk/spm/) running on MATLAB. Each individual 3D-T1 image was coregistered and resliced to its CBF map, and then the coregistered 3D-T1 image was normalized with DARTEL. Subsequently, the transformation matrix was applied to the CBF map.

### FDG-PET Acquisition and Processing

All 27 patients underwent FDG-PET using a combined 16-slice PET/CT scanner (Biograph 16; Siemens, Erlangen, Germany). There were no seizures within the 24 h prior to the examination or during the scan.

After patients had fasted for more than 6 h, their blood glucose levels were measured before the administration of ^18^F-FDG, which involved the intravenous injection of 4–6 MBq/kg ^18^F-FDG 40 min before the start of the brain PET/CT scan. For the emission scans (15 min/bed position; matrix 336 × 336; pixel size, 0.89 × 0.89 mm) of the brain PET/CT protocol (1 bed position; FOV 30.0 cm axial) in 3D mode, a standard PET/CT bed with a built-in head holder was used.

Spatial normalization was performed as for ASL using 3D T1-weighted images, DARTEL, and SPM12.

### Calculation of Regional Values Based on VOIs

Regional values in both ASL and FDG-PET were calculated using PMOD software (http://www.pmod.com/web/), as in our previous publication (21). In this study, we extracted 20 VOIs of the temporal lobe, adjacent cortices, subcortical structures, and cerebellar cortex from the 71 VOIs of the merged version of the Automated Anatomical Labeling template (http://doc.pmod.com/pneuro/aal-mergedatlas6752.html). The 20 VOIs are listed in Table 1. For a reciprocal comparison, the ratio of each VOI to the average accumulation of the whole brain was evaluated. The average accumulation in each participant was calculated by a weighted averaging based on the size of every VOI (21). The calculated regional value (RV) in each VOI represents the ratio of the mean value within the VOI to the mean value of the whole brain. Thus, we calculated each individual's RVs of the 20 VOIs in both FDG-PET and ASL.

**Table 1:** The 20 VOIs used in this study, which were extracted from the 71 VOIs of the AAL-merged Atlas.

**Abbreviation**	**Location**
TL	Temporal, superior, mid, inferior, poles
AMYG	Amygdala
HIP	Hippocampus and parahippocampus
FUSI	Fusiform gyrus
HES	Heschl gyrus
IN	Insula
RO	Rolandic operculum
OLF	Olfactory cortex
SFG	Superior frontal gyrus
MFG	Middle frontal gyrus
IFG	Inferior frontal gyrus
GR	Gyrus rectus
OL	Lateral parts of occipital lobe
SMG	Supramarginal gyrus
ANG	Angular gyrus
CAU	Caudate nucleus
PUT	Putamen
PAL	Pallidum
THAL	Thalamus
CBC	Cerebellum (cerebellar cortex)

We have chosen the VOI analyses based on the following reasons. First, we aimed to investigate “region-level” differences, as our hypothesis suggested. In addition, the detectability of the focus could be well compared by RVs rather than by voxel-wise methods.

### Statistics

To investigate regional side-specific abnormalities in FDG-PET and ASL, we compared the ipsilateral and contralateral RVs in each VOI by a paired *t*-test. As a reference, the average left and right RVs in ASL of healthy controls were calculated and used for the below discrimination analysis. In addition, the ipsilateral RVs in ASL were compared with those of healthy controls by unpaired *t*-test.

Subsequently, we evaluated the detectability of the focus side in the selected VOIs that showed significant side-specific differences in either FDG-PET or ASL. The detectability of the focus was analyzed by receiver operating characteristic (ROC) curves and the area under the curve (AUC). The ROC curves of each VOI were drawn based on the trade-off between sensitivity and specificity for discriminating focus side, and higher AUCs indicates better detectability by RVs of the VOI. The AUCs in each VOI between FDG-PET and ASL were statistically compared (22). In this analysis, the contralateral RVs were used as control values.

Furthermore, we investigated the discriminative power of the ipsilateral RVs from the controls' RVs in ASL alone. We were unable to obtain the normal values of FDG-PET due to ethical concerns. The discriminative analysis in ASL was also performed using ROC curves and the AUC, and the ROC curves were analyzed according to whether the AUC was significantly higher than 0.5 (i.e., random) when differentiating the two conditions (i.e., TLE or controls), based on DeLong et al. (22) and binomial exact methods. In addition, we calculated provisional optimal cut-off values and the sensitivity/specificity for each VOI, based on the Youden index method.

Finally, we have added the ROC curve and AUC analyses with a specific focus on MRI-negative TLE, in consideration of the clinical role of functional neuroimaging in epilepsy. Thus, the ROC curves and AUC analyses were also performed among patients with MRI-negative TLE (*N* = 17) and controls.

For the ROC curve and AUC analyses, we used MedCalc Software version 17.4 (https://www.medcalc.org/). All other analyses were performed with SPSS software, version 23.0 (SPSS Japan, Tokyo, Japan). A *p*-value <0.05 was deemed significant.

## Results

### Clinical Demographics and Visual Inspection

The clinical demographics of the patients with TLE are shown in Table 2. Several patients showed bilateral inter-ictal discharge on conventional scalp EEG, but the focus side was confirmed by video-EEG monitoring. Additionally, the visual analysis of FDG-PET was also compatible in all cases.

**Table 2:** Clinical demographics of patients with unilateral temporal lobe epilepsy in this study.

**No**.	**Age**	**Onset age**	**Epilepsy**	**Seizure**	**IID on EEG**	**VEEG**	**AEDs**	**Etiology on MRI**
1	60–64	9	L-TLE	FIAS, FBTCS	T3	No	PHT, CBZ	L-HS
2	55–59	13	L-TLE	FAS, FIAS	T3	Yes	CBZ, TPM, PRM	Negative
3	35–39	15	L-TLE	FAS, FIAS	F7, T3	No	CBZ, LEV, CZP, NZP	L-HS
4	45–49	29	L-TLE	FIAS	T3	No	CBZ, LTG	Negative
5	25–29	19	L-TLE	FAS, FIAS	T3, T4	Yes	CBZ, LEV, CLB	Negative
6	30–34	24	L-TLE	FIAS	T3	Yes	CBZ, LTG, CZP	Negative
7	45–49	20	L-TLE	FIAS	T3, T4	Yes	CBZ, CZP	Negative
8	35–39	37	L-TLE	FAS, FIAS	T3, T5	No	CBZ	Negative
9	25–29	24	L-TLE	FIAS	T3	No	LEV, VPA	Negative
10	35–39	5	L-TLE	FAS, FIAS	T3	No	CBZ, LTG	L-HS
11	40–44	38	L-TLE	FIAS, FBTCS	T3, T4	Yes	CBZ	Negative
12	30–34	30	L-TLE	FAS, FIAS	T3	Yes	LEV	Negative
13	25–29	4	L-TLE	FAS, FIAS	F7, T1	Yes	CBZ, LEV, LTG	L-HS
14	20–24	20	L-TLE	FIAS, FBTCS	F7, T3	Yes	VPZ, CBZ	Negative
15	50–54	14	R-TLE	FIAS	F8, T4	No	CBZ	R-HS
16	45–49	26	R-TLE	FIAS	T4	No	CBZ, VPA	Negative
17	20–24	3	R-TLE	FAS, FIAS	A2, T4	Yes	LEV, CBZ, PHT	Negative
18	35–39	12	R-TLE	FAS, FIAS, FBTCS	T4, T3	Yes	CBZ, PHT, PRM	R-HS
19	15–19	11	R-TLE	FAS, FIAS	T6	Yes	PHT, LEV, CBZ	Negative
20	35–39	6	R-TLE	FAS, FIAS	T4, F8	Yes	CBZ, LTG	R-HS
21	50–54	11	R-TLE	FAS, FIAS, FBTCS	F8	Yes	CBZ, LTG, LEV	R-HS
22	65–69	40	R-TLE	FIAS	F8	Yes	CBZ, LEV	R-HS
23	40–44	17	R-TLE	FIAS	T4	No	PHT, LTG	Negative
24	35–39	18	R-TLE	FAS, FIAS, FBTCS	T4	Yes	CBZ, LEV	R-HS
25	50–54	22	R-TLE	FIAS	T4, T3	Yes	CBZ	Negative
26	20–29	9	R-TLE	FAS, FIAS	T4, T3	Yes	PHT, LEV, TPM	Negative
27	40–44	20	R-TLE	FIAS	T4	Yes	CBZ, VPA, CLB	Negative

*IID, interictal discharge; VEEG, long-term video-EEG monitoring; AEDs, anti-epileptic drugs; FAS, focal aware seizures; FIAS, focal impaired awareness seizures; FBTCS, focal to bilateral tonic-clonic seizures; CBZ, carbamazepine; PHT, phenytoin; TPM, topiramate; PRM, primidone; LEV, levetiracetam; CZP, clonazepam; CLB, clobazam; NZP, nitrazepam; LTG, lamotrigine; VPA, valproate; HS, hippocampal sclerosis*.

**Figure 1:**
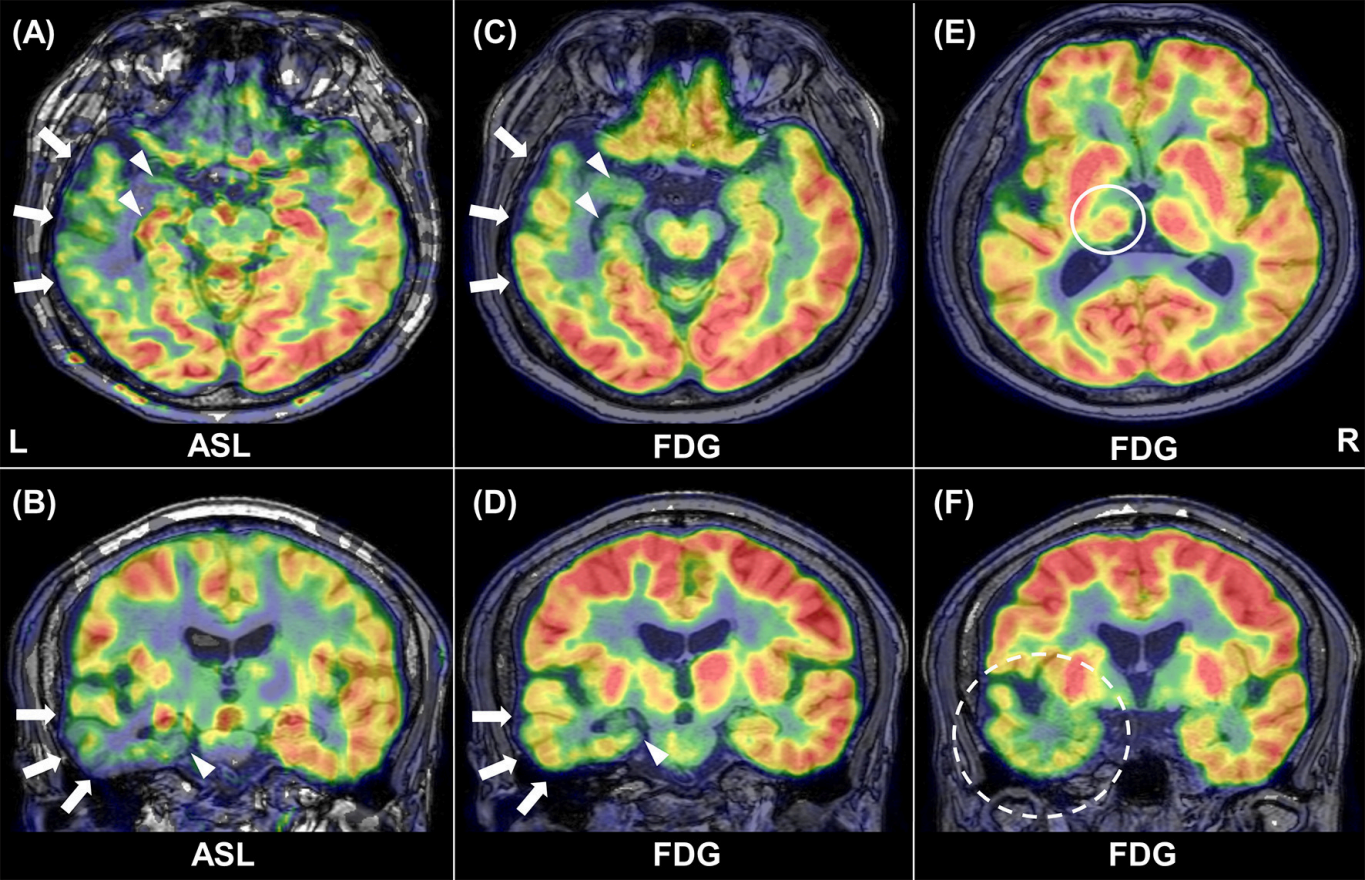
Representative images of ASL and FDG-PET in patient 1. Inter-ictal ASL shows ipsilateral hypoperfusion in the temporal neocortex (arrows) and in the medial temporal lobe (arrowhead) **(A, B)**. Similar findings are present in FDG-PET **(C, D)**. Reduced glucose metabolism can be found in the ipsilateral thalamus [**(E)**, circle] and temporal pole [**(F)**, broken circle].

Example images of FDG-PET and ASL in a patient are shown in Figure 1. Decreased metabolism and CBF were found in widespread areas beyond the ipsilateral mesial temporal lobe.

### Regional Distributions of Significant Differences

**Figure 2:**
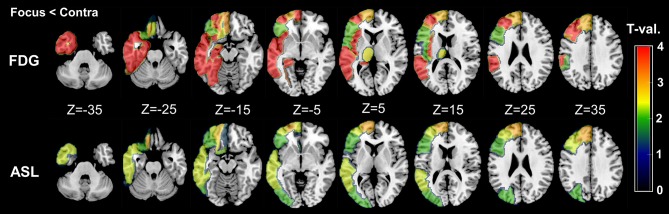
Regional maps of *t*-values (absolute values) in side-specific differences. Only significant VOIs at *p* < 0.05 without Bonferroni correction are shown. For easy understanding, the scale of *t*-values is unified between 0 and 4. However, it should be noted that a few VOIs in FDG-PET showed over 4–5 in the absolute *t*-values (please see also Table 3).

As for the side-specific analyses, the mean RVs for each group and modality are shown in Table 3, and Figure 2 presents regional maps of the *t*-values. Whereas, ASL showed significant side differences mainly in the temporal and frontal lobes, the significant areas in FDG-PET appeared to be more widespread. On the other hand, they only showed trend-level differences in the hippocampus and amygdala in ASL. Both ASL and FDG-PET showed no significance in subcortical structures except the thalamus in FDG-PET. Decreases in the contralateral cerebellum, that is, CCD, were not statistically confirmed with either modality.

**Table 3:** Mean ± SD regional values of both FDG-PET and ASL in patients and of ASL in healthy controls.

**VOIs**	**FDG-TLE**				**ASL-TLE**				**ASL-controls (vs. Ipsi. ASL)**		
	**Ipsi**	**Contra**	***T*-value**	***p*-value**	**Ipsi**	**Contra**	***T*-value**	***p*-value**	**Mean**	***T*-value**	***p*-value**
TL	**0.92 ± 0.05**	**0.99 ± 0.03**	**−5.457**	<0.001^*^	**0.80 ± 0.07**	**0.83 ± 0.06**	**−2.553**	**0.017**	0.85 ± 0.04	**−3.515**	0.001^*^
AMYG	**0.72 ± 0.07**	**0.76 ± 0.05**	**−3.570**	0.001^*^	0.69 ± 0.23	0.76 ± 0.19	−1.841	0.077	0.84 ± 0.09	**−3.250**	**0.003**
HIP	**0.74 ± 0.05**	**0.79 ± 0.04**	**−5.364**	<0.001^*^	0.87 ± 0.16	0.90 ± 0.16	−1.495	0.147	0.93 ± 0.09	−1.792	0.081
FUSI	**0.98 ± 0.04**	**1.02 ± 0.04**	**−5.398**	<0.001^*^	0.87 ± 0.12	0.87 ± 0.10	0.036	0.972	0.88 ± 0.07	−0.552	0.584
HES	**1.21 ± 0.09**	**1.25 ± 0.09**	**−2.749**	**0.011**	1.37 ± 0.16	1.37 ± 0.13	−0.046	0.963	1.37 ± 0.12	−0.019	0.985
IN	**1.01 ± 0.05**	**1.06 ± 0.06**	**−4.915**	<0.001^*^	1.08 ± 0.14	1.10 ± 0.11	−0.905	0.374	1.14 ± 0.08	−1.915	0.063
RO	**1.03 ± 0.05**	**1.05 ± 0.04**	**−2.194**	**0.037**	1.09 ± 0.11	1.10 ± 0.09	−0.438	0.665	1.11 ± 0.08	−0.588	0.558
OLF	0.92 ± 0.04	0.94 ± 0.04	−1.793	0.085	**0.88 ± 0.20**	**0.92 ± 0.19**	**−2.398**	**0.024**	0.98 ± 0.10	**−2.437**	**0.020**
SFG	**0.99 ± 0.04**	**1.01 ± 0.03**	**−3.135**	**0.004**	**0.97 ± 0.08**	**1.00 ± 0.10**	**−3.250**	**0.003**	0.97 ± 0.06	−0.013	0.990
MFG	**1.09 ± 0.04**	**1.11 ± 0.04**	**−4.316**	<0.001^*^	**1.03 ± 0.08**	**1.07 ± 0.10**	**−2.572**	**0.016**	1.03 ± 0.04	0.075	0.940
IFG	**1.01 ± 0.05**	**1.05 ± 0.05**	**−2.309**	**0.029**	**0.91 ± 0.06**	**0.95 ± 0.07**	**−2.210**	**0.036**	0.92 ± 0.04	−1.458	0.152
GR	**1.08 ± 0.05**	**1.10 ± 0.05**	**−2.564**	**0.016**	0.84 ± 0.13	0.86 ± 0.13	−1.142	0.264	0.87 ± 0.07	−1.090	0.283
OL	1.05 ± 0.05	1.07 ± 0.05	−2.019	0.054	**1.00 ± 0.14**	**1.05 ± 0.13**	**−2.194**	**0.037**	1.05 ± 0.09	−1.760	0.083
SMG	**1.02 ± 0.04**	**1.05 ± 0.04**	**−4.144**	<0.001^*^	1.03 ± 0.13	1.06 ± 0.10	−1.051	0.303	1.02 ± 0.06	0.616	0.542
ANG	1.07 ± 0.07	1.12 ± 0.08	−1.908	0.067	1.10 ± 0.09	1.16 ± 0.10	−1.947	0.062	1.13 ± 0.06	−1.273	0.210
CAU	0.93 ± 0.08	0.95 ± 0.07	−1.140	0.265	0.71 ± 0.21	0.70 ± 0.19	0.336	0.740	0.77 ± 0.09	−1.420	0.165
PUT	1.25 ± 0.06	1.25 ± 0.07	−0.177	0.861	0.90 ± 0.18	0.89 ± 0.16	0.463	0.647	0.88 ± 0.07	0.392	0.698
PAL	0.99 ± 0.08	1.00 ± 0.06	−0.885	0.384	0.72 ± 0.23	0.72 ± 0.20	−0.189	0.851	0.74 ± 0.09	−0.340	0.736
THAL	**1.02 ± 0.07**	**1.05 ± 0.06**	**−2.712**	**0.012**	1.05 ± 0.23	1.06 ± 0.26	−0.395	0.696	1.14 ± 0.13	−1.856	0.071
CBC	0.89 ± 0.05	0.89 ± 0.05	0.298	0.768	0.87 ± 0.12	0.86 ± 0.10	0.206	0.838	0.86 ± 0.06	0.417	0.678

*The abbreviations of the VOIs are explained in Table 1. Bold font denotes significant side-differences at uncorrected p <0.05*.

* *denotes significance at p <0.05 after Bonferroni correction for 20 comparisons*.

**Figure 3:**
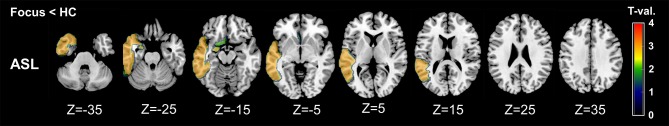
Regional maps of *t*-values (absolute values) in comparison between ipsilateral side and mean healthy control values. Only significant VOIs at *p* < 0.05 without Bonferroni correction are shown.

On the other hand, compared with RVs of healthy controls, ipsilateral RVs showed significant decreases mainly in temporal neocortex and amygdala (Table 3 and Figure 3).

### Detectability of the Focus Side in FDG-PET and ASL

The ROC-AUC analysis was performed for 15 VOIs. The AUC values and the comparison between FDG-PET and ASL are shown in Table 4. In general, detectability was higher in FDG-PET, with the difference statistically significant in four VOIs, including the temporal lobe structures and insula (Table 4).

**Table 4:** Detectability of the focus side compared with contralateral values as control.

	**All cases (*****N*** **= 27)**			**MRI-negative only (*****N*** **= 17)**		
**VOIs**	**FDG-AUC**	**ASL-AUC**	***p***	**FDG-AUC**	**ASL-AUC**	***p***
TL	**0.874**	**0.652**	**0.006**	0.806	0.664	0.159
AMYG	0.649	0.623	0.792	0.540	0.595	0.671
HIP	**0.775**	**0.558**	**0.009**	0.692	0.578	0.212
FUSI	**0.785**	**0.521**	0.003^*^	0.727	0.595	0.267
HES	0.660	0.501	0.162	0.630	0.561	0.641
IN	**0.726**	**0.534**	**0.047**	0.740	0.561	0.170
RO	0.638	0.558	0.488	0.578	0.664	0.549
OLF	0.595	0.591	0.971	0.564	0.529	0.816
SFG	0.657	0.616	0.631	0.761	0.626	0.277
MFG	0.643	0.619	0.783	0.689	0.626	0.593
IFG	0.664	0.678	0.872	0.633	0.616	0.873
GR	0.620	0.531	0.426	0.588	0.509	0.571
OL	0.571	0.591	0.841	0.526	0.640	0.392
SMG	0.727	0.624	0.340	0.713	0.734	0.876
THAL	0.632	0.512	0.171	0.564	0.554	0.925

*The AUC values of ROC curves and the comparison between FDG-PET and ASL are shown. The abbreviations of the VOIs are explained in Table 1. Bold font denotes significant differences at uncorrected p < 0.05 between FDG-PET and ASL*.

* *denotes significance at p < 0.05 after Bonferroni correction for 15 comparisons*.

### Discriminative Power From Control Values in ASL

The AUC values in three VOIs showed significant discriminative power in ASL (Table 5). The VOIs of the temporal cortex and amygdala showed moderate AUC values (~0.75, *p* < 0.001 for both VOIs).

**Table 5:** Discriminative power of ipsilateral ASL values compared with healthy controls.

	**All cases (*****N*** **= 27)**					**MRI-negative only (*****N*** **= 17)**				
**VOIs**	**ASL-AUC**	***p***	**Cut-off**	**Sens**.	**Spec**.	**ASL-AUC**	***P***	**Cut-off**	**Sens**.	**Spec**.
TL	**0.740**	<0.001^*^	**≤0.82**	**66.7**	**78.4**	**0.798**	<0.001^*^	**≤0.78**	**58.82**	**94.59**
AMYG	**0.769**	<0.001^*^	**≤0.68**	**55.6**	**100.0**	**0.733**	**0.015**	**≤0.68**	**58.82**	**100.0**
HIP	0.615	0.145	≤0.82	40.7	94.6	0.609	0.248	≤0.82	35.3	94.6
FUSI	0.561	0.442	≤0.83	48.2	78.4	0.591	0.330	≤0.83	52.9	78.3
HES	0.514	0.861	≤1.52	77.8	5.4	0.533	0.720	≤1.28	35.3	81.2
IN	0.616	0.127	≤1.02	29.6	97.3	0.630	0.142	≤1.06	47.1	78.4
RO	0.561	0.426	≤1.13	74.1	46.0	0.649	0.073	≤1.13	88.2	46.0
OLF	**0.660**	**0.033**	**≤0.87**	**48.2**	**91.9**	0.609	0.273	≤0.79	41.2	94.6
SFG	0.515	0.853	≤0.90	25.9	91.9	0.626	0.174	≤0.90	41.2	91.9
MFG	0.519	0.825	≤0.98	37.0	91.9	0.628	0.197	≤0.97	47.1	94.6
IFG	0.584	0.281	≤0.86	33.3	94.6	0.582	0.382	≤0.84	29.4	97.3
GR	0.572	0.383	≤0.78	37.0	94.6	0.558	0.581	≤0.80	47.1	83.4
OL	0.626	0.091	≤0.97	40.7	86.5	0.610	0.259	≤0.97	47.1	86.5
SMG	0.517	0.840	≤1.13	74.1	0.0	0.612	0.241	≤1.01	70.6	64.9
THAL	0.649	0.055	≤1.02	55.6	86.5	0.644	0.121	≤1.02	52.9	86.5

*The AUC values of ROC curves and the sensitivity/specificity at the optimal cut-off value are shown. The abbreviations of the VOIs are explained in Table 1. Bold font denotes statistically significant AUC values at uncorrected p < 0.05*.

* *denotes significance at p < 0.05 after Bonferroni correction for 15 comparisons*.

### Detectability of the Focus Side in MRI-Negative TLE Only

The AUC values compared with contralateral side or healthy controls are shown in Tables 4, 5. The results showed similar tendencies to those from all cases. However, the differences between FDG-PET and ASL were not statistically significant (Table 4).

## Discussion

In the current study, we compared the regional distributions of FDG-PET and ASL in TLE. Although both modalities showed decreased values in similar areas, including the temporal and frontal cortices, we found more widespread areas with decreases in FDG-PET, including the ipsilateral thalamus, insula, supramarginal gyrus, and medial temporal structures. Similarly, the detectability of the focus side compared with the contralateral side was generally higher in FDG-PET. The detectable values in ASL were relatively higher in VOIs from the temporal neocortex and amygdala. However, at this stage, it seems that ASL couldn't present comparable clinical usefulness with FDG-PET.

Recently, increasing numbers of studies have reported the usefulness of inter-ictal ASL for focus detection in both TLE (6, 7, 9, 13, 14) and extratemporal focal epilepsy (7, 8, 10–12). However, given the uncoupling of CBF and glucose metabolism in epilepsy (18), the distributions of ASL and FDG-PET would not be expected to overlap exactly. The lower sensitivity of inter-ictal perfusion SPECT than FDG-PET (4) would support this speculation. Therefore, it is expected and compatible with previous knowledge that we found similar and differing regional distributions and different detectabilities between FDG-PET and ASL.

Another possible explanation for our findings would be the larger dispersion of ASL. Judging from Table 3, the mean side differences were not very different between the modalities in several VOIs, with the higher SD in ASL possibly affecting the statistical significance. ASL imaging may be subject to the effects of noise or arteries, even with a median filter technique. Additionally, according to a previous study (11), ASL can detect hyperperfusion in the focus even beyond 24 h after seizures. In addition, the optimal timing for ASL scanning after seizures is still not established, and this may have resulted in the larger dispersion of ASL values compared with FDG-PET.

Previous articles on ASL for epilepsy have reported significant asymmetry or concordance with focus by using visual assessment (7, 11) or VOIs mainly in the medial temporal lobe (6, 9, 13, 14). The sensitivity of ASL for focus detection was previously reported as 74% (11), which appears almost concordant with our results in the temporal lobe VOI (Table 5). According to a previous study, FDG-PET and ASL showed high concordance for the lateralization or lobe-level localization of the focus, but the correlations in various brain regions were not necessarily significant (10). Our study focused on the regional distributions of abnormality between the two modalities, and the results deepen our understanding and contribute to further applications of ASL imaging.

We also investigated subcortical structures and the cerebellum. The ipsilateral thalamus showed a significantly decreased value in FDG-PET, but not in ASL. On the other hand, there was no side difference in the cerebellum on either FDG-PET or ASL, probably because CCD is more often seen in frontal or parietal lobe epilepsy (4). Although the extensive glucose hypometabolism in remote areas is supposed to be related to longstanding uncontrolled seizures involving propagation pathways (4), the findings in ASL had not been sufficiently investigated. In this respect, we found an uncoupling of CBF and glucose metabolism in the ipsilateral thalamus, and further investigations that include extratemporal focal epilepsy may provide significant insights into the diaschisis of metabolism and CBF.

Furthermore, we added the sub-group analyses with a specific focus on MRI-negative TLE. Whereas, structural MRI is a strong tool to detect the focus of TLE, there exist cases with no visible lesion on MRI. However, even in such MRI-negative TLE, functional neuroimaging (e.g., FDG-PET) can provide significant information of focus side (23) as well as favorable surgical outcome (24). Thus, MRI-negative TLE is one of the important targets of FDG-PET and ASL imaging. Based on the current results, the regional AUC values showed similar patterns to those from all cases, although the statistical significance was not evident probably due to the reduced sample size.

Several efforts have been made to improve ASL imaging technique. In TLE, pulsed arterial spin labeling (pASL) was reported to show better lateralization than dynamic susceptibility contrast enhanced (14). PCASL, which was used in this study, is a more advanced method using trains of rapid radiofrequency pulses and alternating bipolar magnetic field gradients (25). It is known that pCASL has higher precision and signal-to-noise ratio than standard pASL or continuous ASL (25). ASL imaging is still being developed, which could possibly provide significant progress to the field of focal epilepsy in the future. However, more fundamentally, we might have to consider whether inter-ictal perfusion imaging can inherently surpass FDG-PET or not, given the lower sensitivity of inter-ictal perfusion SPECT (4). At any rate, we may consider that ASL is currently not comparable with FDG-PET and still needs further advances for clinical use.

This study has several limitations. First, our cohort is not a large sample and might include neocortical TLE in addition to medial TLE, which may have affected the results, particularly in medial temporal VOIs including the hippocampus. However, the results in FDG-PET were robust and generally consistent with medial temporal onset. Another limitation could be the non-simultaneous acquisition of FDG-PET and ASL. However, the reproducibilities of the two examinations are high (26, 27) and robust correlations between PET/MR and PET/CT have also been reported (10). Finally, the accumulated alpha errors due to multiple VOI analyses must be kept in mind. We found that some of results were not rigorously significant with Bonferroni correction. On the other hand, the main aim of this study was not to demonstrate a rigorous abnormality in the focus of TLE, but to investigate regional distributions of both modalities. In addition, we also found more robust results in several VOIs, and thus we believe these findings will deepen our knowledge of ASL imaging.

## Conclusion

In TLE, both ASL and FDG-PET showed decreased values in similar areas, including the ipsilateral temporal and frontal cortices, but a more widespread and severe decrease was found in FDG-PET in the ipsilateral thalamus, insula, supramarginal gyrus, and medial temporal structures. The detectability of the focus side was generally higher in FDG-PET. The detectable values in ASL were relatively higher temporal neocortex and amygdala VOIs. At this stage, it seems that ASL couldn't present comparable clinical usefulness with FDG-PET. The results further our understanding of ASL imaging and will contribute to its more widespread application.

## Data Availability

The datasets generated for this study are available on request to the corresponding author.

## Author Contributions

DS organized the whole study. DS, NS, and HM were involved in the study concept and design. DS, YK, and MO performed recruitment and data acquisition. NM analyzed data. DS wrote the manuscript. All authors read and approved the submitted version.

### Conflict of Interest Statement

The authors declare that the research was conducted in the absence of any commercial or financial relationships that could be construed as a potential conflict of interest.
